# LuHui Derivative, A Novel Compound That Inhibits the Fat Mass and Obesity-Associated (FTO), Alleviates the Inflammatory Response and Injury in Hyperlipidemia-Induced Cardiomyopathy

**DOI:** 10.3389/fcell.2021.731365

**Published:** 2021-11-22

**Authors:** Ying Yu, Yumiao Pan, Ziyi Fan, Silun Xu, Zhiyuan Gao, Zijing Ren, Jie Yu, Wen Li, Fangtong Liu, Jintao Gu, Ye Yuan, Zhimin Du

**Affiliations:** ^1^ Institute of Clinical Pharmacology, The Second Affiliated Hospital of Harbin Medical University (University Key Laboratory of Drug Research, Heilongjiang Province), Harbin, China; ^2^ Department of Clinical Pharmacology, College of Pharmacy, Harbin Medical University, Harbin, China; ^3^ State Key Laboratory of Quality Research in Chinese Medicines, Macau University of Science and Technology, Macau, China

**Keywords:** hyperlipidemia, inflammation, LuHui derivative, FTO, CD36, m^6^A

## Abstract

Hyperlipidemia is a major risk factor for metabolic disorders and cardiovascular injury. The excessive deposition of saturated fatty acids in the heart leads to chronic cardiac inflammation, which in turn causes myocardial damage and systolic dysfunction. However, the effective suppression of cardiac inflammation has emerged as a new strategy to reduce the impact of hyperlipidemia on cardiovascular disease. In this study, we identified a novel monomer, known as LuHui Derivative (LHD), which reduced the serum levels of total cholesterol (TC), triglycerides (TG), low-density lipoprotein cholesterol (LDL-C), and reduced lipid deposition in cardiomyocytes. In addition, LHD treatment improved cardiac function, reduced hyperlipidemia-induced inflammatory infiltration in cardiomyocytes and suppressed the release of interleukin-6 (IL-6) and tumor necrosis factor-α (TNF-α). From a mechanistic perspective, cluster of differentiation 36 (CD36), an important cell surface receptor, was identified as a downstream target following the LHD treatment of palmitic acid-induced inflammation in cardiomyocytes. LHD specifically binds the pocket containing the regulatory sites of RNA methylation in the fat mass and obesity-associated (FTO) protein that is responsible for elevated intracellular m^6^A levels. Moreover, the overexpression of the N6-methyladenosine (m^6^A) demethylase FTO markedly increased CD36 expression and suppressed the anti-inflammatory effects of LHD. Conversely, loss-of-function of FTO inhibited palmitic acid-induced cardiac inflammation and altered CD36 expression by diminishing the stability of CD36 mRNA. Overall, our results provide evidence for the crucial role of LHD in fatty acid-induced cardiomyocyte inflammation and present a new strategy for the treatment of hyperlipidemia and its complications.

## 1 Introduction

Dyslipidemia causes damage to multiple tissues and organs and is a critical risk factor for atherosclerotic cardiovascular disease (Liu et al., 2017). Hyperlipidemia is characterized by the accumulation of total cholesterol (TC), triglycerides (TG), and low-density lipoprotein cholesterol (LDL-C). Increasingly, the evidence suggests that chronic systemic hyperlipidemia contributes to nonalcoholic fatty liver disease, diabetes, muscle and heart tissue damage, and the inflammatory response (Nicholls and Lundman, 2004; Ertunc and Hotamisligil, 2016; Healy et al., 2016; Ralston et al., 2017).

The heart is a non-adipose organ in which fatty acids mainly contribute to myocardial ATP production. The remaining ATP is provided by glucose and lactate, making the heart sensitive to lipotoxicity. Fatty acid metabolism disorders and lipid accumulation in cardiomyocytes result in the release of various cytokines, especially promoting the secretion of leukocyte adhesion molecules and chemokines, culminating in cardiomyocyte damaged and impaired diastolic and systolic functions (Schilling et al., 2012; Jia et al., 2016). Recent studies have examined the novel traditional Chinese medicines that may prevent the progression of hyperlipidemia (Bian et al., 2020; Chen et al., 2020; Su et al., 2020).

It has been recently reported that the dysregulation of m^6^A methylation is associated with adipogenesis, the innate immune response, and many diseases (Liu et al., 2019; Wang L. et al., 2020). As the first regulatory factor of the m^6^A modification, FTO was shown to exert a protective effect against alcohol-induced liver inflammation (Yu et al., 2020). FTO was also shown to play an essential role in the regulation of food intake and adipose synthesis (Hess et al., 2013; Ben-Haim et al., 2015). However, it has not been confirmed if FTO is a promising target for the treatment of hyperlipidemia-induced cardiomyocyte inflammation.

CD36 acts as a multifunctional membrane protein facilitating long-chain fatty acid uptake. It interacts with the membrane receptor Toll-like receptor 4 (TLR4), to co-regulate the oxidized low density lipoprotein (oxLDL)-induced inflammatory responses in the cardiac and skeletal muscle (Stewart et al., 2010; Sheedy et al., 2013). Owing to this functionality, CD36 has been confirmed as a promising candidate to alter the link between myocardial fatty acid utilization and the regulation of the inflammatory response, particularly in hyperlipidemia (Silverstein et al., 2010).

The aims of this study were to investigate the effect of a novel synthetic anthraquinone compound, LuHui Derivative (LHD, chemical name: 1,8-dihydroxy-3-(hydroxymethyl)-anthraquinone ethyl succinate), against hyperlipidemia-induced cardiomyocyte inflammation and to reveal the underlying molecular mechanisms of the modification of FTO and CD36. These results confirmed that LHD is a potential novel compound for the treatment of hyperlipidemia-induced cardiac inflammation.

## 2 Materials and Methods

### 2.1 Chemicals and Reagents

**FIGURE 1:**
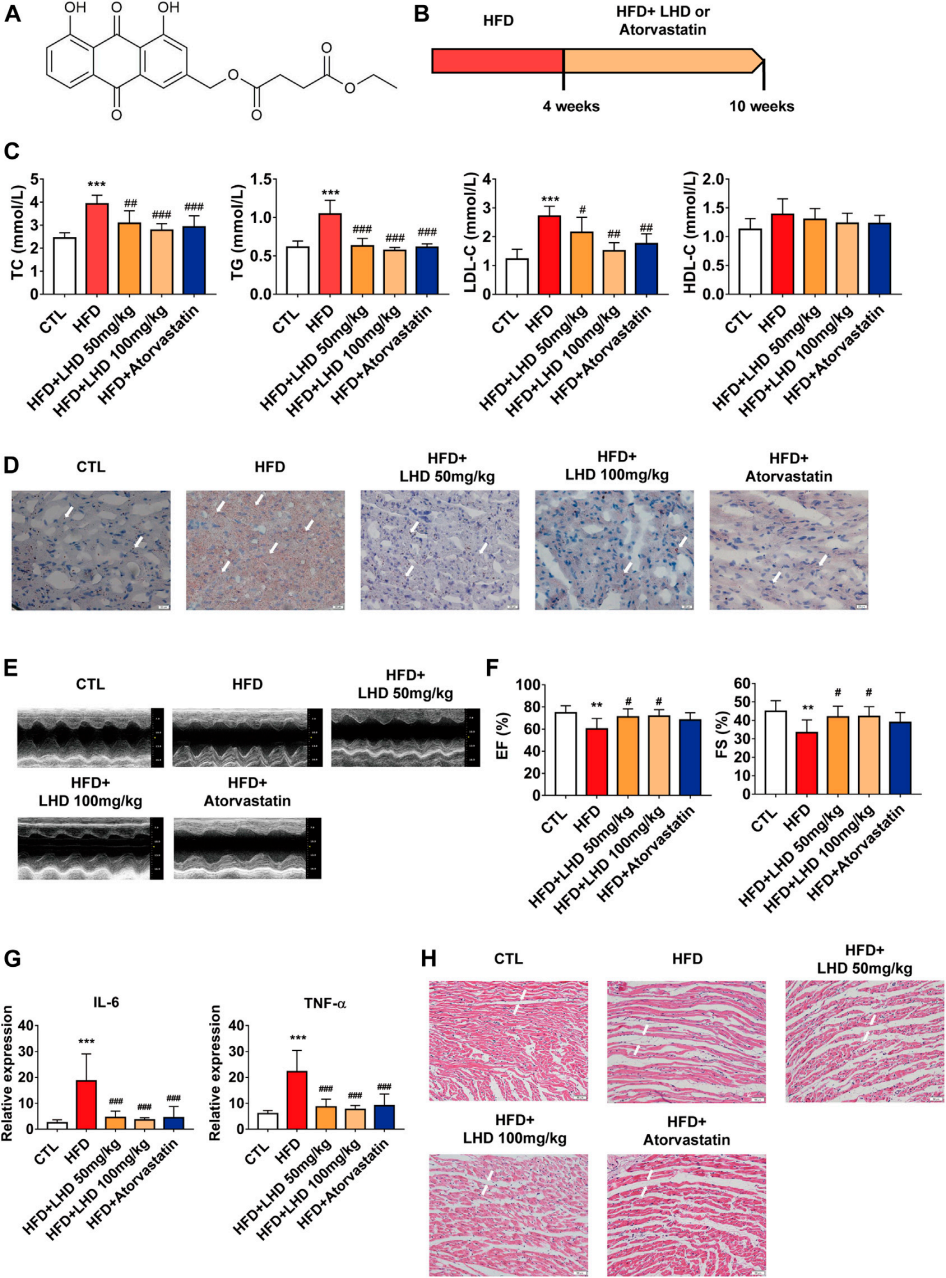
LHD protects against hyperlipidemia-induced cardiac dysfunction. **(A)** Chemical structure of LHD. **(B)** Wistar rat feeding schedule. **(C)** Measurement of TC, TG, LDL-C, and HDL-C in the serum of rats fed a high-fat diet (HFD) for 10 weeks. ***p < 0.001, compared with the CTL group; ^#^ *p* < 0.05, ^##^ *p* < 0.01, ^###^ *p* < 0.001, compared with the HFD group, *n* = 8. **(D)** Oil Red O staining to identify lipid deposition in cardiac tissue (10× magnification). *n* = 4. **(E,F)** Echocardiographic analysis of left ventricular ejection fraction (EF%) and left ventricular shortening fraction (FS%) in rats fed with HFD for 10 weeks. **p < 0.01, compared with the CTL group; ^#^ *p* < 0.05, compared with the HFD group, *n* = 8. **(G)** ELISA analysis of IL-6 and TNF-α in the serum. ***p < 0.001, compared with the CTL group; ^###^ *p* < 0.001, compared with the HFD group. *n* = 8. **(H)** H&E staining to identify infiltrative inflammation in cardiac tissue (10× magnification), *n* = 4.

The LHD monomer (purity > 95%) (Figure 1A), provided by the Department of Pharmaceutical and Chemical Research, Harbin Medical University, was dissolved in dimethyl sulfoxide (DMSO). Palmitic acid (PA) was purchased from Sigma (St. Louis, MO) and dissolved in 0.1 N sodium hydroxide and 15% bovine serum albumin (BSA), filtered, and then stored at -20°C. The high-fat diet (HFD) feed, consisting of 77.6% maintenance feed, 10% lard, 10% yolk powder, 2% cholesterol, 0.2% bile salt, and methylthiouracil 0.2%, were purchased from HuaFuKang Biotechnology (Beijing, China).

### 2.2 Animals

The animal procedures in this study were approved by the Animal Experimental Ethics Committee of Harbin Medical University. To avoid the complicating effects of estrogen on cardiovascular disease (Sabbatini and Kararigas, 2020; Meng et al., 2021), healthy male Wistar rats (180–220 g), purchased from the Experimental Animal Center of the Affiliated Second Hospital of Harbin Medical University (Harbin, China), were used in the study. The rats were maintained on a HFD for 4 weeks to establish hyperlipidemia, which was determined by analyzing the serum levels of TC, TG, LDL-C, and high-density lipoprotein cholesterol (HDL-C) in blood samples from orbital region of rats (Supplementary Figure S1). Following the method of previous studies (Su et al., 2020), the rats were divided into four groups: HFD, HFD with LHD 50 mg kg^−1^, HFD with LHD 100 mg kg^−1^, and HFD with atorvastatin 7.2 mg kg^−1^ (Figure 1B). In control (CTL) group, an equal volume of distilled water was administered. Heart tissue and serum samples were collected after 6 weeks of continuous daily intragastric administration for the corresponding follow-up experiments.

### 2.3 Cell Culture

Human cardiomyocyte AC16 cell line was kindly gifted by Prof. Ben-zhi Cai (Department of Pharmacy at The Second Affiliated Hospital) and grown in DMEM containing 10% fetal bovine serum (Biological Industries, Israel), 1% penicillin, and 1% streptomycin at 37°C in a humidified atmosphere containing 5% CO_2_ (v v^−1^). Cells were treated with LHD (25 µM) for 30 min and then added PA (500 µM) for 16 h to establish an *in vitro* model of inflammatory response induced by HFD.

### 2.4 Cell Transfection

For transfection, AC16 cells were grown to 60–70% confluence. We used Lipofectamine RNAiMAX Reagent (Thermo Fisher Scientific, CA, United States, 13778075) to transfect small interfering RNAs (siRNAs) and Lipofectamine 3,000 (Thermo Fisher Scientific, CA, United States, 3000015) to transfect plasmids for gene overexpression. The transfection was performed in accordance with the manufacturer’s instructions (Han et al., 2021). Briefly, 2 µl targeting siRNA or negative control (NC) siRNA was mixed with 150 µl Opti-MEM (Thermo Fisher Scientific, CA, United States, 31985070) and 9 µl Lipofectamine RNAiMAX reagent was mixed with 150 µL Opti-MEM together at room temperature. For the overexpression analysis, the pHG-CMV-Kan2-FTO plasmid (Yingrun Biotechnology, Changsha, China, HG-HO080432) and pHG-CMV-Kan2-CD36 plasmid (Yingrun Biotechnology, Changsha, China, HG-HO000072) were diluted and transfected into AC16 cells. After 24 or 48 h of transfection, the cells were harvested for analysis. The siRNA sequences are shown in Supplementary Table S1.

### 2.5 Quantitative Real-Time Polymerase Chain Reaction (qRT-PCR)

Total RNA was isolated from the heart tissue and cells by using Trizol reagent (Invitrogen, Carlsbad, CA, United States); 0.5–1 µg of RNA was used to prepare cDNA using the Reverse Transcription Kit (Vazyme Biotech, Nanjing, China, R223-01) in accordance with the manufacturer’s protocol. qRT-PCR was performed in a 10 µl volume with SYBR Green PCR Master Mix (Roche, Switzerland, RC-4913914001). The qRT-PCR analysis was performed on a LightCycler^®^ 480 II (Roche, Switzerland) comprising initial denaturation, annealing, and extension steps. The real-time PCR conditions were: denaturation at 95°C for 10 s, followed by 40 cycles of 95°C for 10 s and 55°C for 30 s. The sequences of the specific primers used for qRT-PCR are shown in Supplementary Table S2.

### 2.6 Enzyme-Linked Immunosorbent Assay (ELISA)

Abdominal aortic blood was collected and centrifuged at 3,000 rpm for 15 min at 4°C to gather serum samples. To verify the protein expression of TNF-α and IL-6 in rat serum, the rat TNF-α ELISA kit (ABclonal, Wuhan, China, ab208348) and the rat IL-6 ELISA kit (ABclonal, Wuhan, China, ab100712), respectively, were used in accordance with the kit instructions. The supernatant was centrifuged at 1,000 rpm for 5 min prior to the analysis.

### 2.7 Cellular Thermal Shift Assay (CETSA)

The CETSA experiment was based on previously reported methods (Martinez Molina et al., 2013; Jafari et al., 2014). Briefly, after 100 µM LHD treatment for 3 h, the cells were collected in PBS and centrifuged at 300 g for 5 min, and the supernatant was discarded. Aliquots of the cells were transferred to separate Eppendorf tubes, subjected to a temperature gradient, and kept at room temperature for 3 min. After three freeze-thaw cycles, the supernatant was centrifuged and analyzed by sodium dodecyl sulfate-polyacrylamide gel electrophoresis (SDS-PAGE).

### 2.8 Drug Affinity Responsive Target Stability (DARTS) Assay

The DARTS assay was performed in accordance with a previously reported method (Lomenick et al., 2009; Pai et al., 2015). Briefly, we divided the cells into three groups: control (CTL), LHD, and input. First, we centrifuged the cells at 18,000 g for 10 min at 4°C in lysis solution. An appropriate volume of 10× TNC was added to the supernatant to detect the protein concentration using the bicinchoninic acid assay. Cell lysates were incubated with 83.4, 166.7, and 250.0 µM LHD at room temperature for 1 h before pronase digestion. Finally, the lysates were analyzed by western blotting to determine the binding between LHD and FTO.

### 2.9 Protein Extraction and Western Blotting

Western blotting analysis was performed as described in the previous report (Zhao et al., 2021). The cells were lysed in lysis buffer. After centrifugation, the supernatant was collected and the protein concentration was quantified by BCA assay kit. The samples were boiled at 95°C for 5 min and immediately frozen in a –80°C refrigerator. The primary antibodies were used as follows: anti-CD36 (ABclonal, Wuhan, China, A5792, 1:1,000), anti-GAPDH (ABclonal, Wuhan, China, AC033, 1:1,000), anti-FTO (ABclonal, Wuhan, China, A1438, 1:1,000), anti-P65-S536 (ABclonal, Wuhan, China, AP0475, 1:1,000), anti-IKB-α (Proteintech, Wuhan, China, 10268-1-AP, 1: 1,000), and anti-β-actin (ABclonal, Wuhan, China, AC026, 1: 1,000).

### 2.10 Echocardiography

The Wistar rats were anesthetized by intraperitoneal injection with avertin and the hair near the chest area was removed, as previously described (Cai et al., 2020). The cardiac function and heart dimensions were determined by two-dimensional echocardiography. Echocardiography was performed using a Vevo 1,100 VisualSonics device (VisualSonics, Toronto, ON, Canada). The left ventricular ejection fraction (EF%) and left fractional ventricular shortening (FS%) were calculated using M-mode images.

### 2.11 Histology

The heart tissue was fixed with 4% paraformaldehyde, embedded in paraffin, and sectioned into 5 μM coronal slides for further analysis. Hematoxylin eosin (H&E) staining (Solarbio, Beijing, China, g1260) was performed according to the method recommended by the manufacturer.

### 2.12 Oil Red O Staining

The tissues were fixed with paraformaldehyde and cryosectioned. The slides were first rinsed with PBS to wash off the embedding medium, and then soaked with 60% isopropyl alcohol. Staining with Oil Red O (Solarbio, Beijing, China, G1120) before washing with 60% isopropyl alcohol until the background was colorless. After counterstaining with hematoxylin, the slides were sealed with glycerol-gelatin.

### 2.13 Dot Blot

To determine the level of m^6^A in cells, the dot blot assay were performed on total RNA or poly (A)^+^ RNA, as described previously (Nagarajan et al., 2019). In brief, AC16 cells were treated with LHD (25 µM) for 16 h and transfected with FTO siRNA for 24 h before the dot blot assay. The RNA samples were diluted in RNase-free buffer, denatured at 95°C for 5 min, and immediately cooled, and then crosslinked by UV irradiation following stained with methylene blue (Solarbio, Beijing, China, G1300). After incubating with 5% skim milk, the membrane was detected with m^6^A antibody (ABclonal, Wuhan, China, A19841, 1:1,000). Finally, the membrane was analyzed using an Odyssey (LICOR Biosciences, Lincoln, NE, United States).

### 2.14 Statistical Analysis

All data were expressed as mean ± standard deviation of mean unless otherwise noted. Data analysis was performed using GraphPad Prism 7.0 software. The significance of the differences was analyzed using Studentʼs *t*-test or one-way analysis of variance (*p < 0.05, **p < 0.01, ***p < 0.001 compared with the CTL group, ^#^ *p* < 0.05, ^##^ *p* < 0.01, ^###^ *p* < 0.001 compared with the HFD or PA group). *p* < 0.05 was considered statistically significant.

## 3 Results

### 3.1 LuHui Derivative Protects Against HFD-Induced Inflammation

To determine the role of LHD (Figure 1A) in hyperlipidemia and the associated cardiac function, we employed an HFD-induced rats model of hyperlipidemia. The *in vivo* experimental protocol is shown in Figure 1B. First, we analyzed the serum concentrations of TC, TG, LDL-C, and HDL-C in Wistar rats fed on a HFD for 4 weeks. Compared with the CTL group, the circulating concentrations of TC, TG, and LDL-C were higher in the HFD group (Supplementary Figure S1). After 6 weeks of continuous treatment with LHD, the serum levels of TC, TG, and LDL-C were significantly reduced, but there were no significant change in HDL-C levels. The same changes were observed in the atorvastatin treatment group (Figure 1C). Next, we performed Oil Red O staining to analyze lipid aggregation in the heart, and found that the administration with HFD for 10 weeks led to the accumulation of excess lipids in cardiomyocytes; however, treatment with LHD or atorvastatin significantly alleviated this accumulation (Figure 1D). Moreover, cardiac echocardiography showed that, compared with the CTL group, the administration of HFD clearly decreased the EF% and FS%; in contrast, LHD treatment significantly mitigated the hyperlipidemia-induced cardiac systolic and diastolic dysfunction (Figures 1E,F). These results indicated that LHD treatment altered the hyperlipidemia-associated changes in lipids and cardiac function *in vivo*.

Hyperlipidemia-induced dysfunction is usually accompanied by a spontaneous inflammatory response in cardiomyocytes (Wang Y. et al., 2017). Therefore, we performed an ELISA to detect the expression of pro-inflammatory cytokines. As shown in Figure 1G, hyperlipidemia resulted in higher serum concentrations of TNF-α and IL-6, but LHD and atorvastatin treatment significantly reduced the expression of inflammatory factors. Furthermore, H&E staining revealed that LHD and atorvastatin treatment reduced infiltrative inflammation in the heart of HFD-fed rats (Figure 1H). These results implied that LHD effectively reduced the inflammation associated with hyperlipidemia.

### 3.2 LuHui Derivative Protects Against Cardiomyocyte Inflammation *In Vitro*

**FIGURE 2:**
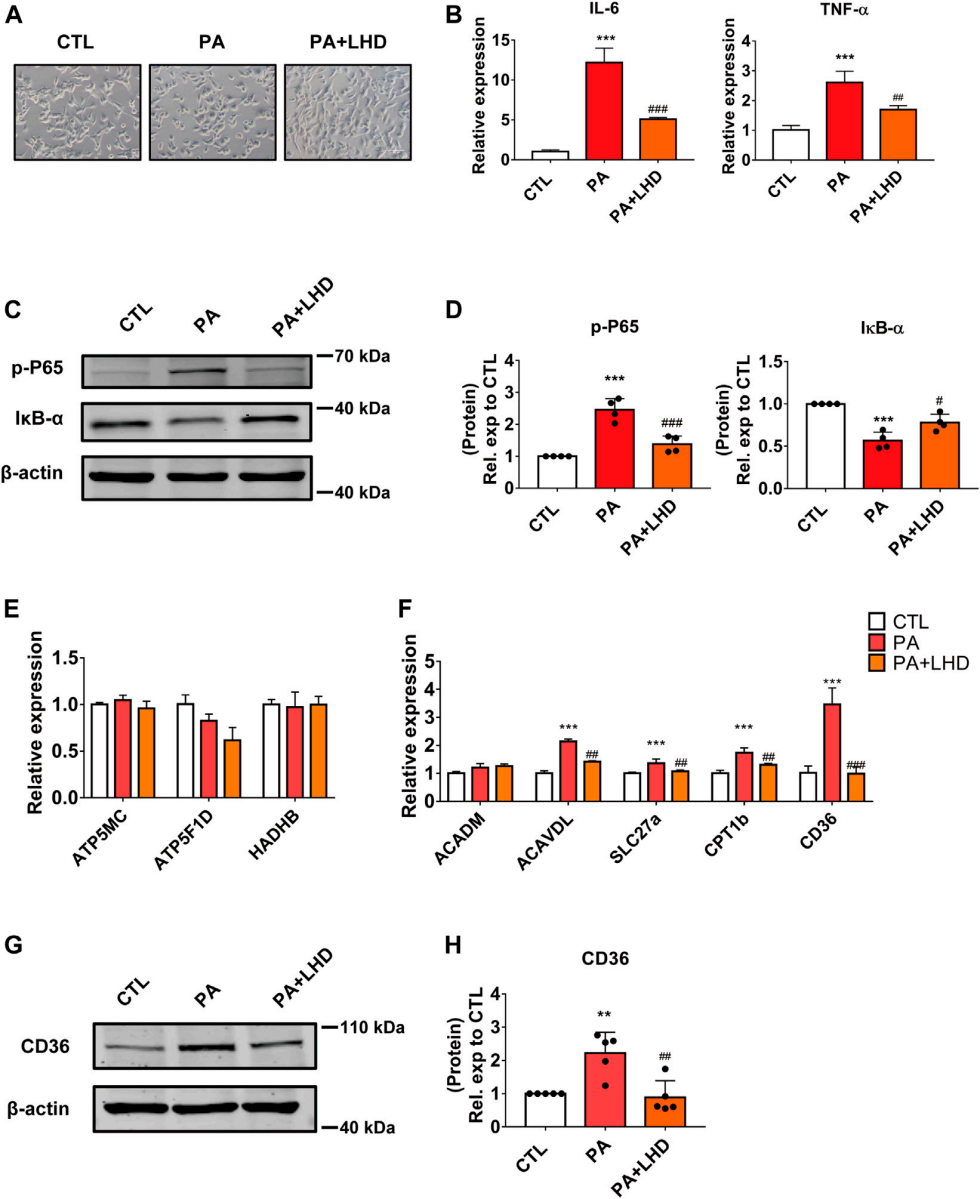
LHD inhibits PA-induced cardiomyocyte inflammation *in vitro*. **(A)** Representative images of cell morphology captured by phase microscopy (20× magnification). *n* = 3. **(B)** Effect of LHD treatment on pro-inflammatory gene expression in AC16 cells treated with PA for 16 h. ***p < 0.001, compared with the CTL group, ^##^ *p* < 0.01, ^###^ *p* < 0.001, compared with the PA group, *n* = 3. **(C,D)** Changes in the intracellular inflammatory signaling pathway after LHD and PA treatment. **(C)** Western blotting analysis of the expression of the p-P65 and IκB-α inflammatory signaling proteins in human cardiomyocytes; **(D)** Statistical analysis of protein expression, ***p < 0.001, compared with the CTL group; ^#^ *p* < 0.05, ^###^ *p* < 0.001, compared with the PA group, *n* = 4. **(E,F)** Cardiac mRNA expression of **(E)** oxidative phosphorylation-related enzymes and **(F)** fatty acid metabolism-related genes. ***p < 0.001, compared with the CTL group; ^##^ *p* < 0.01, ^###^ *p* < 0.001, compared with the PA group, *n* = 3. **(G,H)** Western blotting analysis of the regulatory effect of LHD on CD36 expression. **p < 0.01, compared with the CTL group; ^##^ *p* < 0.01, compared with the PA group, *n* = 5.

Hyperlipidemia and other lipid metabolic diseases induce inflammation that are predominantly associated with saturated fatty acids. Of the various fatty acids, PA (16:0) plays an essential role in this process (Liu et al., 2017). To detect the anti-inflammatory effect of LHD *in vitro*, we stimulated AC16 cardiomyocytes with PA to mimic hyperlipidemia-induced inflammation. First, we observed the cell morphology after PA and LHD treatment. We found that, compared with the CTL group, PA treatment led to cardiomyocyte more rounded, but this was alleviated by PA treatment followed by LHD treatment (Figure 2A). Moreover, PA treatment stimulated the mRNA expression of the proinflammatory factors IL-6 and TNF-α, and LHD treatment significantly reduced this abnormal elevation (Figure 2B). However, LHD prevented the PA-induced phosphorylation and degradation of NF-κB P65 and IκB-α (Figures 2C,D).

Cardiomyocyte damage caused by hyperlipidemia disrupts the balance between metabolic enzymes and fatty acids (Son et al., 2018). To determine whether LHD rescued the hyperlipidemia-induced inflammatory phenotype by altering metabolism-related enzyme activity, we investigated the expression of oxidative phosphorylation-related enzymes and fatty acid metabolism enzymes in cardiomyocytes. The results showed that PA stimulation increased the expression of most metabolism-related enzymes; the most significant change occurred in CD36 expression and this increase was notably inhibited by LHD treatment (Figures 2E,F). Consistent with this, Western blot dected that CD36 expression was clearly suppressed in LHD-treated cells (Figures 2G,H). Collectively, these data indicate that CD36 may play an important role in the LHD-mediated regulation of the inflammatory response in cardiomyocytes.

### 3.3 CD36 Regulates PA-Induced Inflammation

**FIGURE 3:**
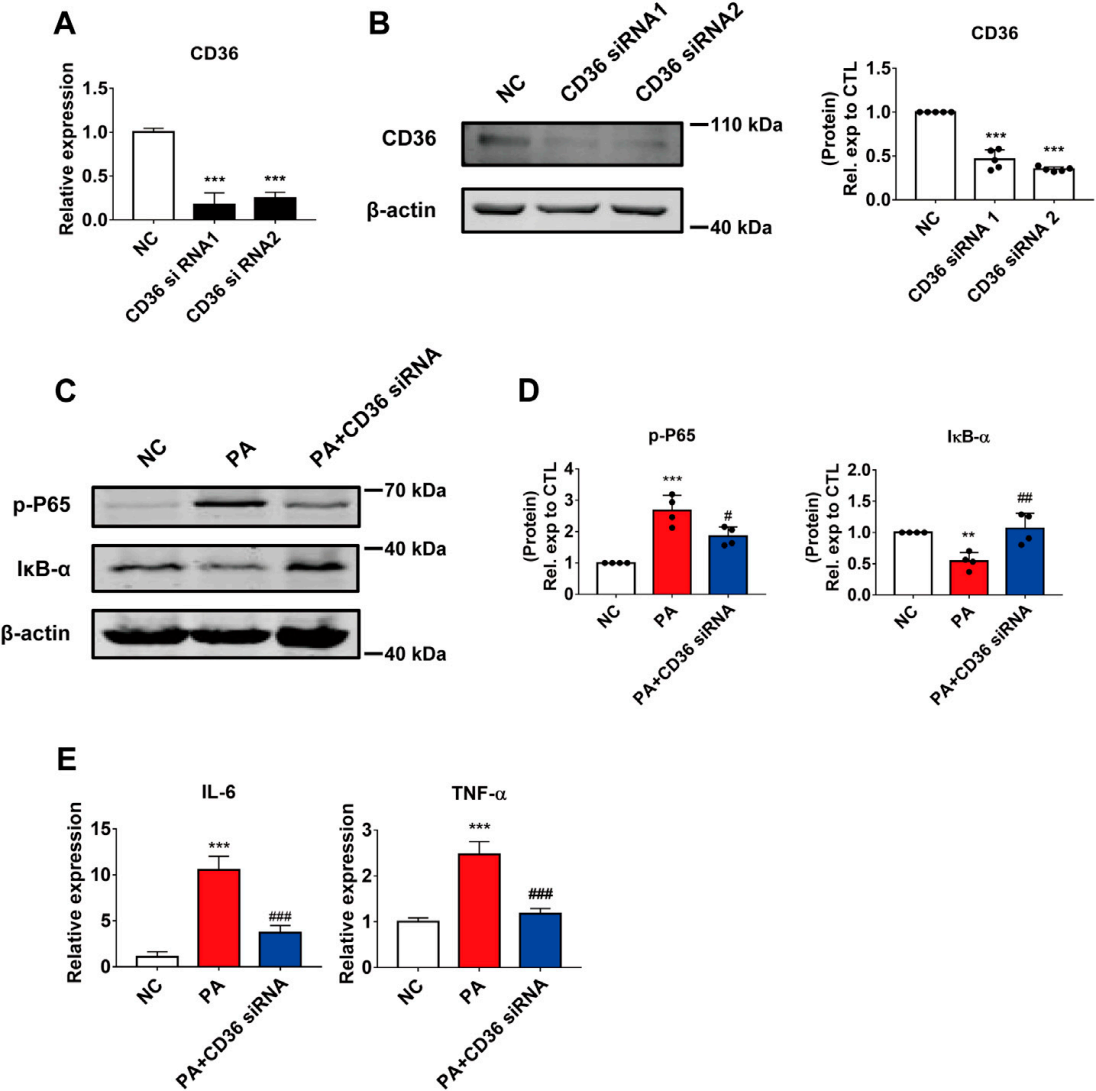
The effect of CD36 in PA-induced cardiomyocyte inflammation. **(A,B)** Silencing efficiency of CD36. **(A)** qRT-PCR analysis of the effect of transfection of CD36 siRNA after 24 h in AC16 cells. ***p < 0.001, compared with the negative control (NC) group, *n* = 3. **(B)** Western blotting analysis of the effect of CD36 siRNA transfection after 48 h in AC16 cells. ***p < 0.001, compared with the NC group, *n* = 5. **(C,D)** Changes in the intracellular inflammatory signaling pathway after CD36 siRNA and PA treatment. **(C)** Western blotting analysis of the expression of the p-P65 and IκB-α proteins in human cardiomyocytes; **(D)** Statistical analysis of protein expression, **p < 0.01, ***p < 0.001 compared with the NC group; ^#^ *p* < 0.05, ^##^ *p* < 0.01, compared with the PA group, *n* = 4. **(E)** qRT-PCR analysis of the expression of the inflammatory genes IL-6 and TNF-α in CD36 siRNA and PA-treated cells. ***p < 0.001, compared with the NC group; ^###^ *p* < 0.001, compared with the PA group, *n* = 4.

To further investigate the effect of CD36 in PA-induced cardiomyocyte inflammation, we used siRNA to silence the expression of CD36 in AC16 cells. Two CD36 siRNA oligomers were tested and significant reductions were achieved in the mRNA and protein expression; siRNA2 was selected for subsequent studies (Figures 3A,B). As expected, the effect of silencing CD36 was similar to that of LHD treatment: the reduced expression of CD36 restored NF-κB phosphorylation and IκB-α expression in PA-treated cells (Figures 3C,D; Supplementary Figure S2A). Moreover, qRT-PCR results suggested that the expression of pro-inflammatory factors was markedly reduced by CD36 loss of function (Figure 3E). To further verify the important role of CD36 in LHD anti-PA-induced cardiac inflammation, we transfected the exogenous CD36 plasmid into AC16 cells. qRT-PCR analysis showed that CD36 overexpression significantly interfered with the mRNA expression of CD36, IL-6 and TNF-α rescued by LHD (Supplementary Figure S2B–D). These results suggest that CD36 is required for the LHD anti-PA stimulation of cardiomyocytes.

### 3.4 LuHui Derivative Binds to FTO and Inhibits its Activity

**FIGURE 4:**
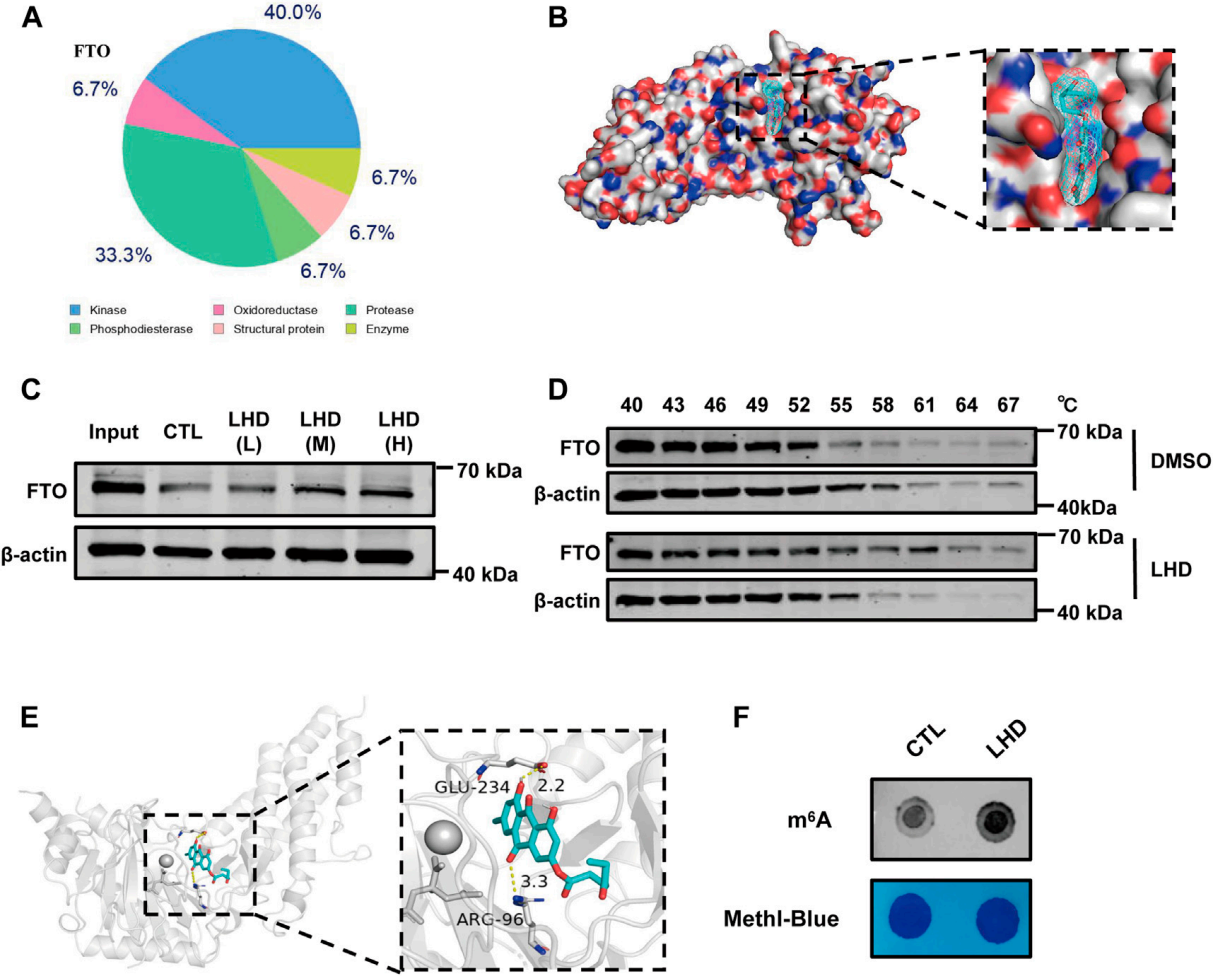
LHD binds the FTO protein and regulates the level of m^6^A modifications. **(A)** The top 15 compounds most likely to bind LHD. **(B)** Docking models simulating LHD binding into the FTO crystal structure (PDB ID: 3LFM). **(C)** Representative western blotting analysis of FTO from the DARTS assay. AC16 cells lysates with 83.4 (LHD L), 166.7 (LHD M), and 250.0 (LHD H) µM LHD were incubated for 1 h at room temperature before pronase digestion, *n =* 3. **(D)** Western blotting analysis of the thermal stability of FTO in LHD-treated cells, *n* = 3. **(E)** Structure of the complex of FTO bound with LHD; the yellow dotted lines indicate hydrogen bonding. **(F)** Determination of the abundance of the modification in 25 µM LHD-treated cells after 16 h, *n* = 4.

Next, to explore the underlying anti-inflammatory mechanism of LHD in cardiomyocytes, we used SwissTargetPrediction, a small molecule compound and protein binding prediction website, to predict potential binding partners of LHD. We found that FTO, as an adiposity and obesity-related gene and an adipose metabolism-related gene (Wang et al., 2015), was the most promising LHD-binding candidate and may play a regulatory role in PA-induced inflammation process (Figure 4A; Supplementary Table S3). To understand the binding of LHD and FTO, we performed molecular docking studies of LHD and the FTO protein using AutoDockTools 1.5.6 and PyMol 2.4 software. We found that LHD bound directly within the FTO catalytic pocket (Figure 4B). In addition, CETSA and DARTS assays were used to confirm the binding between LHD and FTO *in vitro*. As expected, the FTO protein resisted pronase activity in a dose-dependent manner in the presence of LHD (Figure 4C), and direct binding with LHD increased the thermal stability of the FTO protein (Figure 4D).

Notably, we found that expression of the FTO protein expression was not altered by the PA-induced inflammatory response or the anti-inflammatory effect of LHD (Supplementary Figure S3A,B). We then investigated whether LHD disrupted the enzymatic action of FTO. We observed the structure of LHD bound to FTO and found that LHD binds specifically to amino acids R96 and E234 of FTO (Figure 4E). The accumulated evidence shows that the N atom on the m^6^A purine ring interacts with the R96 and E234 residues of the FTO protein via H-bonding, which locks the m^6^A base in place (Zhang et al., 2019). To confirm these findings, we assessed whether LHD regulated FTO-mediated m^6^A demethylation in cells. We performed a m^6^A dot blot to analyze mRNA and found that m^6^A methylation was increased in LHD-treated cells (Figure 4F; Supplementary Figure S3C). Overall, these results suggested that LHD effectively bonded with the FTO protein and inhibited m^6^A demethylation by FTO.

### 3.5 FTO Regulates CD36 Expression in PA-Induced Cardiomyocyte Inflammation

**FIGURE 5:**
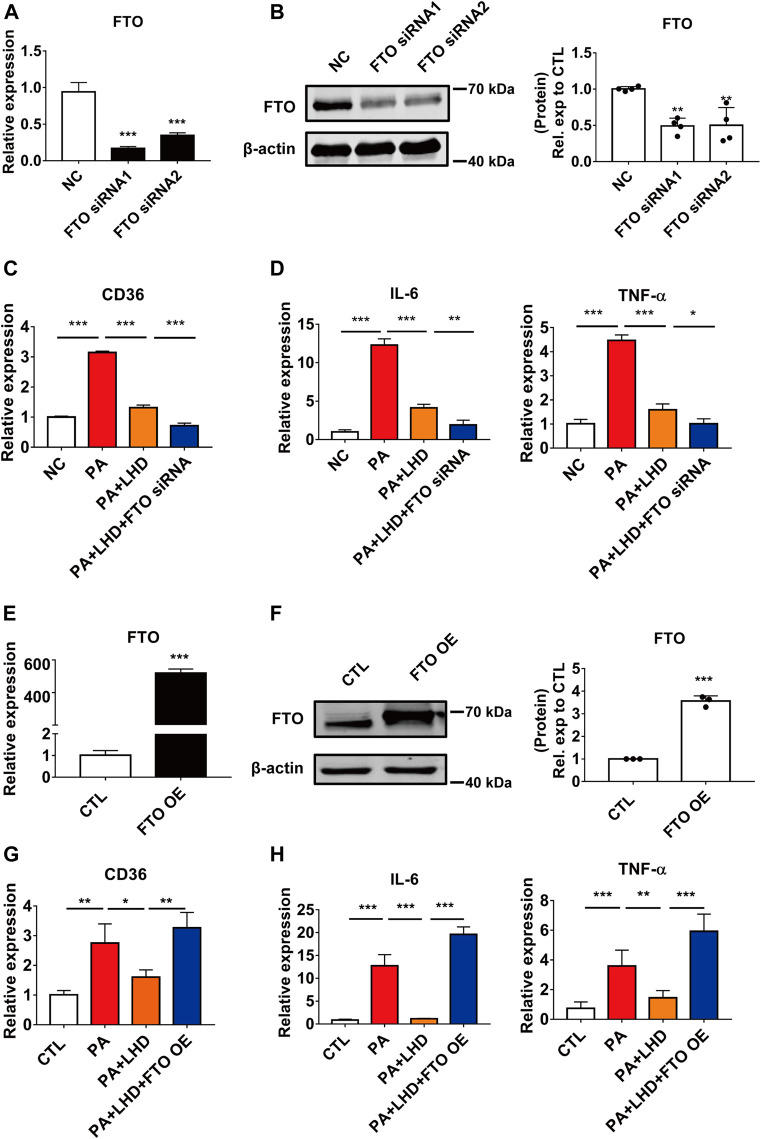
FTO alters CD36 expression. **(A,B)** Silencing efficiency of FTO. **(A)** qRT-PCR analysis of the effect of transfection of FTO siRNA after 24 h in AC16 cells. ***p < 0.001, compared with the NC groups, *n* = 3. **(B)** Western blotting analysis of the effect of transfection of FTO siRNA after 48 h in AC16 cells. **p < 0.01, compared with the NC group, *n* = 4. **(C)** Effect of FTO loss of function and LHD co-treatment on CD36 expression in AC16 cells. ***p < 0.001, *n* = 3. **(D)** Effect of FTO loss of function and LHD co-treatment on IL-6 and TNF-α expression in AC16 cells. *p < 0.05, **p < 0.01, ***p < 0.001, *n* = 3. **(E,F)** Efficiency of FTO overexpression. **(E)** Expression of FTO mRNA after FTO overexpression for 24 h in AC16 cells. **(F)** Expression of FTO protein after FTO overexpression for 48 h in AC16 cells. ***p < 0.001, compared with the CTL group, *n* = 3. **(G)** Effect of FTO overexpression on the expression of CD36 in PA-treated AC16 cells. *p < 0.05, **p < 0.01, *n* = 3. **(H)** Effect of FTO overexpression on the expression of IL-6 and TNF-α in PA-treated AC16 cells. **p < 0.01, ***p < 0.001, *n* = 3.

To further investigate the effect of FTO on the PA-induced inflammatory response and CD36 expression, we used siRNA to silence intracellular FTO expression. The qRT-PCR and western blotting results showed that the two siRNAs tested, which targeted different regions of FTO, significantly reduced FTO expression, and the more effective siRNA, siRNA1, was selected for the subsequent experiments (Figures 5A,B; Supplementary Figure S4A). As reported in previous studies, FTO silencing significantly enhanced intracellular levels of the m^6^A modification (Supplementary Figure S4B). The loss of function of FTO significantly enhanced the inhibitory effect of LHD in CD36 mRNA expression (Figure 5C), and the mRNA expression of IL-6 and TNF-α was markedly suppressed by FTO siRNA treated cells (Figure 5D; Supplementary Figures S4C–E).

To verify the effect of FTO in LHD against PA-induced cardiac inflammation, we transfected exogenous FTO plasmid and confirmed that the mRNA and protein expression of FTO was significantly increased (Figures 5E,F). From the qRT-PCR analysis, we found that the mRNA expression of CD36, IL-6 and TNF-α was significantly restored in LHD-treated cells. However, this was altered by FTO overexpression (Figures 5G,H), Moreover, the FTO overexpression plasmid significantly enhanced intracellular CD36 protein expression (Supplementary Figure S4F), which indicated that FTO is required to mediate the anti-inflammatory effects of LHD in PA-treated cardiomyocytes.

### 3.6 FTO-Dependent m^6^A Modification Regulates CD36 Stability

**FIGURE 6:**
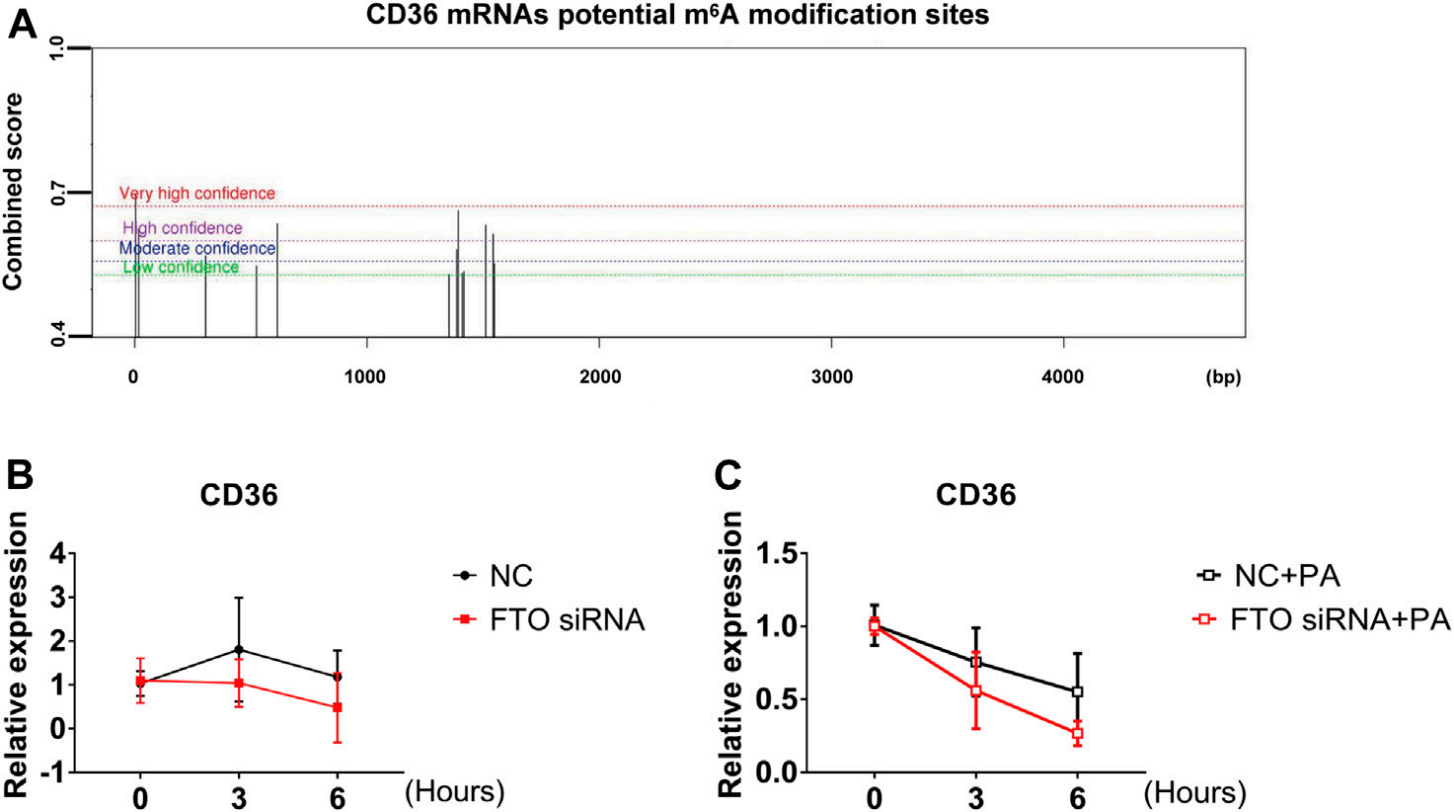
FTO regulates CD36 expression through the m^6^A modification. **(A)** The prediction of potential m^6^A modification sites on CD36 mRNA. **(B)** Stability of CD36 mRNA in AC16 cells after FTO siRNA transfection for 24 h. **(C)** qRT-PCR analysis of CD36 mRNA stability after FTO silencing in PA-treated stimulation cells, *n* = 3.

It was shown that m^6^A binding proteins selectively recognize the dynamic m^6^A modification to regulate the stability of mRNA and the translation status (Wang et al., 2014). In combination with the above experiments showing that LHD, as an FTO inhibitor, regulates intracellular m^6^A levels and mitigates the PA-induced increase in CD36 mRNA expression. Therefore, we hypothesized that the m^6^A modification regulates the expression of CD36 in cardiomyocytes through its impact on mRNA transcription. To verify this hypothesis, we predicted the association between the m^6^A modification sites and CD36 transcript by SRAMP, a sequence-based predictor of m^6^A modification sites. The results showed that there were many m^6^A modification sites on the CD36 transcript (Figure 6A). Next, we determined the stability of CD36 mRNA after cells were transfected with FTO siRNA. We found that the half-life of CD36 mRNA was not significant change in FTO expression (Figure 6B). Thus, we examined the stability of CD36 mRNA following PA-induced inflammation. As expected, FTO silencing significantly decreased the half-life of CD36 mRNA compared with PA treatment (Figure 6C). Together, these results indicate that LHD/FTO-mediated m^6^A demethylation impairs the stability of CD36 mRNA and protects against fatty acid-induced inflammation.

## 4 Discussion

**FIGURE 7:**
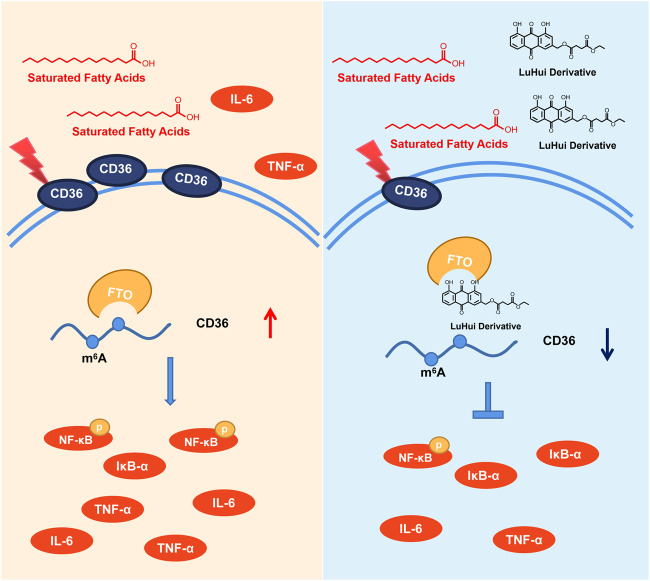
Underlying mechanisms of the protective effect of LHD against hyperlipidemia-induced cardiomyocyte inflammation.

Herein, we have reported a novel chemically modified monomer, LHD, with protective effects against hyperlipidemia-induced cardiac injury, and determined the molecular mechanisms of these protective effects. Our results showed that LHD clearly reduced the lipid serum levels and alleviated cardiac inflammation *in vitro* and *in vivo* models of hyperlipidemia. Moreover, LHD decreased the expression of the inflammatory cytokines IL-6 and TNF-α *in vitro* model of PA stimulation (Figure 7).

Current knowledge suggests that the inflammation induced by a long-term HFD is directly associated with a range of metabolic diseases, including hyperlipidemia, insulin resistance, and type 2 diabetes (Goldberg et al., 2012). These diseases usually result in cardiac metabolic disorders, cardiomyocyte hypertrophy, apoptosis, fibrosis, inflammation, and systolic dysfunction (Palomer et al., 2013; Steven et al., 2018). Thus, maintaining of systemic metabolic homeostasis is a promising strategy to preserve heart function. In this study, we found that LHD had beneficial effects on cardiac function and alleviated inflammation by regulating CD36 expression and inhibiting the release of pro-inflammatory factors. In addition, this study provided persuasive evidence that LHD was an encouraging candidate for the treatment of cardiovascular-related metabolic diseases.

FTO is involved in the development of many metabolic diseases (Sun et al., 2020). In our study, we found that suppressing the expression of FTO inhibited the PA-induced inflammatory response in cardiomyocytes. The connection between FTO and cardiac disease has also been studied recently. Susmita demonstrated the importance of FTO-dependent m^6^A methylation for cardiac systolic function and suggested that the overexpression of FTO reduced fibrosis in a mice model of myocardial infarction (MI) (Mathiyalagan et al., 2019). Moreover, Berulava et al. (2020) demonstrated that cardiac-specific FTO knockdown in transgenic mice delayed heart recovery and exacerbated heart failure. These experiments demonstrated the negative regulatory role of FTO in cardiomyocytes. However, these diseases were not associated with fatty acid metabolism or lipid accumulation. Clinical studies have confirmed fundamental differences in the mechanism of cardiometabolic disorders, obesity, and lipotoxicity-induced cardiovascular disease compared with ischemic cardiovascular disease (Obokata et al., 2017). In addition, research consistent with this finding showed that the endothelial cell-specific knockdown of FTO protected against obesity-induced metabolic disorders and insulin resistance, and improved cardiac functions (Kruger et al., 2020). The complex role of FTO in different cardiovascular diseases indicates that it has an essential role in fatty acid metabolism disorder-induced heart injury, as well as provides an effective therapeutic strategy for the utilization of FTO inhibitors in metabolic diseases.

Our research demonstrated that LHD was an FTO inhibitor and regulated the inflammatory responses in cardiomyocytes. This evidence supported further exploration of FTO inhibitors and expands the potential therapeutic applications for FTO. The non-steroidal anti-inflammatory drug meclofenamic acid (MA) binded specifically to FTO and inhibits FTO enzyme activity (Huang et al., 2015). Subsequently, Huang added a five-membered heterocycle to the structure of MA, showing that the novel compounds FB23 and FB23-2 were effective for the treatment of acute myeloid leukemia owing to their inhibition of the FTO methylesterase (Huang et al., 2019). Likewise, we showed that the chemically modified monomeric compound LHD could bind to FTO and interfere with the FTO-mediated m^6^A modification, thereby ameliorating the hyperlipidemia- and PA-induced inflammatory responses in cardiomyocytes. These findings have enriched the body of knowledge concerning FTO inhibitors and provides a theoretical basis for further applications of FTO application.

Interestingly, we detected the expression of m^6^A methylation modifying enzymes during PA pro-inflammatory and LHD anti-inflammatory processes. The results showed that PA treatment significantly reduced the changes in the expression levels of METTL3 and ALKBH5, but did not affect the intracellular expression levels of FTO and METTL14. During the anti-inflammatory process of LHD, the expression of m^6^A methyltransferase (METTL3 and METTL14) and demethylase (ALKBH5 and FTO) did not change (Supplementary Figure S3A,B). This result suggested that LHD does not specifically affect the expression of methylation-modifying enzymes in PA-induced inflammation rescued by LHD. In regard to another FTO inhibitor, rhein, was shown to suppress FTO function in myogenic differentiation while not affecting FTO expression (Wang X. et al., 2017). Furthermore, FTO loss-of-function markedly affected intracellular m^6^A modification and the release of inflammatory cytokines. The simultaneous overexpression of FTO enhanced the PA-induced inflammatory responses. Consistent with this finding, in the LPS-treated intestinal porcine epithelial J2 (IPEC-J2) cells and human dental pulp cells (HDPCs), FTO expression was not significantly altered during the inflammatory response, but the regulation of m^6^A modification alleviated the inflammatory response (Feng et al., 2018; Zong et al., 2019). In atherosclerosis, a chronic inflammatory disease of arteries, FTO expression was not altered, but increased m^6^A modification also influenced the development of atherosclerosis (Terpenning et al., 1987; Zhang et al., 2020). Based on these findings, we proposed several hypotheses: 1) The process of PA-induced inflammation is complex, and FTO may be sensitive to the substrates and mRNA maintain stability; 2) The binding of LHD in the FTO pocket only affects the m^6^A demethylation ability of FTO, and does not induce FTO protein degradation; 3) The primary function of FTO is demethylation.

The m^6^A modification, the most abundant type of mRNA modification, is involved in many processes, including mammalian development, immunity, stem cell renewal, fat differentiation, tumorigenesis, and metastasis (Geula et al., 2015; Zhao et al., 2017; Li et al., 2018; Weng et al., 2018; He et al., 2019; Winkler et al., 2019; Song et al., 2020). It has also been shown that m^6^A has an essential role in the formation and accumulation of fat. During these processes, the expression of CD36 decreases as the expression of FTO was decreased (Heng et al., 2020). Similarly, the level of m^6^A modification was also affected by thermal stimulation, and CD36 was consistently elevated with FTO in pig abdominal fat. However, the regulatory relationship between FTO and CD36 has not previously been reported.

The m^6^A methylation recognition protein, YTHDF2, has been shown to play essential roles, including the regulation of adipocyte autophagy, in a multitude of FTO-regulated diseases, such as alcohol-induced renal inflammation (Wu et al., 2018; Wang X. et al., 2020). The knockdown of YTHDF2 markedly increased the MAPK and NF-κB signaling pathways activity, increasing expression of pro-inflammatory cytokines and exacerbating inflammation in LPS-stimulated RAW 264.7 cells (Yu et al., 2019). Moreover, the knockdown of YTHDF2 in hepatoma cells inhibited the release of inflammatory cytokines and reduced macrophage clearance of hepatoma cells (Hou et al., 2019). These results demonstrated that YTHDF2 plays a critical role in the intracellular regulation of inflammatory factors. Therefore, it is reasonable to assume that FTO regulates the stability of CD36 mRNA via YTHDF2 dependent on the m^6^A modification in hyperlipidemia and PA-induced cardiomyocyte inflammation.

Several studies indicated that CD36 may contribute to cardiac dysfunction and heart failure in metabolic disorders with an inflammatory background; the factors regulating CD36 expression were well studied. It was well established that peroxisome proliferator-activated receptor (PPAR) combines with retinoid X receptor (RXR) binding in the CD36 transcription region in ox-LDL or under transforming growth factor-β (TGF-β) stimulation (Han et al., 2000; Yu et al., 2016). Moreover, the promoter region of CD36 also could bind with a variety of regulators, including pregnane X receptor (PXR) reaction components, the liver X receptor (LXR), and the CCAAT/enhancer-binding protein (C/EBP) (Zhou et al., 2008). This observation was consistent with previous findings showing that signal transducer and activator of transcription 3 (STAT3) combine in an interferon-γ-activated sequence (GAS) in the CD36 promoter region to promote angiogenesis, tumor invasion, and metastasis (Sp et al., 2018). Similar outcomes, namely that microRNAs directly regulated CD36 expression, including miR-4668 and miR-26a, have been reported (Li et al., 2017; Ding et al., 2019). However, little is known regarding the contribution of the m^6^A modification to CD36 expression. Our study found that the FTO-mediated m^6^A modification alters CD36 expression by impairing the stability of CD36 mRNA. This finding will expand the known influence of m^6^A and provide a basis for the further research into CD36.

However, the limitations of our study should be considered. First, we found the binding of LHD and FTO *in vivo* using published detection methods, but we could not conclusively determine whether LHD is involved in modifying proteins other than FTO. Second, the clinical development of hyperlipidemic cardiomyopathy requires prolonged exposure. Experimentally, although acute or short-term exposure to saturated fatty acids is adequate for research purposes, it may not encompass all the complications of abnormal lipid metabolism. In addition, owing to the availability of hyperlipidemic samples, we were unable to perform complete analyses of the experimental rats and cultured cells. Although essential measurements were collected in human cardiomyocytes for a better understanding of the clinical applications of LHD, further studies using samples from patients with hyperlipidemia would be necessary.

In the future, it is likely that research into inflammation will progress beyond conventional areas, to novel fields such as cardiomyocyte reprogramming. Our study has contributed to a deeper understanding of the inflammatory processes and provides a potential direction for the application of traditional Chinese anti-inflammatory medicines. With the continuous development of science and technology and the discovery of protein structures, the binding relationships and regulatory mechanisms between small molecule compounds and proteins can be studied in more depth. Such studies have led to clear progress in understanding the regulatory relationships between protein molecules and their applications in medicinal research.

## 5 Conclusion

Our study has demonstrated that LHD protects against hyperlipidemia-induced myocardial injury and PA-induced cardiomyocyte inflammation. Mechanistically, LHD binds to the FTO-specific m^6^A binding site, inhibiting the demethylation modification of FTO, and suppressing the expression of the CD36, which consequently regulates myocardial metabolism and suppresses inflammation. This study has revealed the therapeutic effect of FTO on the heart and provides a platform to support the development of FTO inhibitors.

## Data Availability

The original contributions presented in the study are included in the article/Supplementary Material, further inquiries can be directed to the corresponding authors.
