# Supplementary material for: LuHui Derivative, A Novel Compound That Inhibits the Fat Mass and Obesity-Associated (FTO), Alleviates the Inflammatory Response and Injury in Hyperlipidemia-Induced Cardiomyopathy
Source: Front Cell Dev Biol. 2021 Nov 22;9:731365. doi: 10.3389/fcell.2021.731365 (PMC8647038; doi:10.3389/fcell.2021.731365)
Supplement: Supplementary file 1 [file DataSheet1.docx]

# Supplementary data


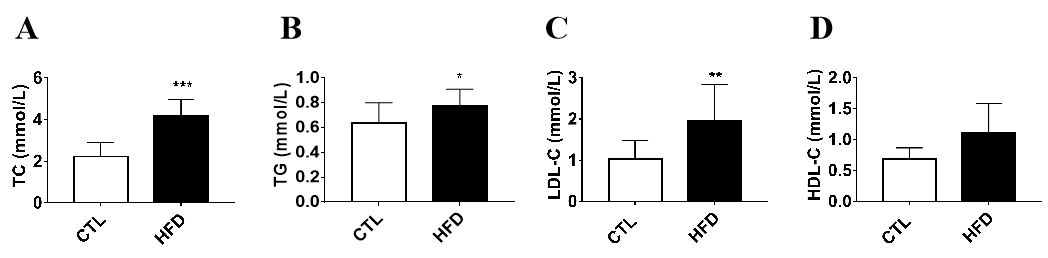


**Supplementary Figure 1：Lipid content of rats at 4 weeks.**

**(A-D)** Detect (A) TC, (B) TG, (C) LDL and (D) HDL in rat serum at 4 weeks, ** *p* <0.01, *** *p* <0.001, compared with the CTL group, CTL *n* = 8, HFD *n* = 32.


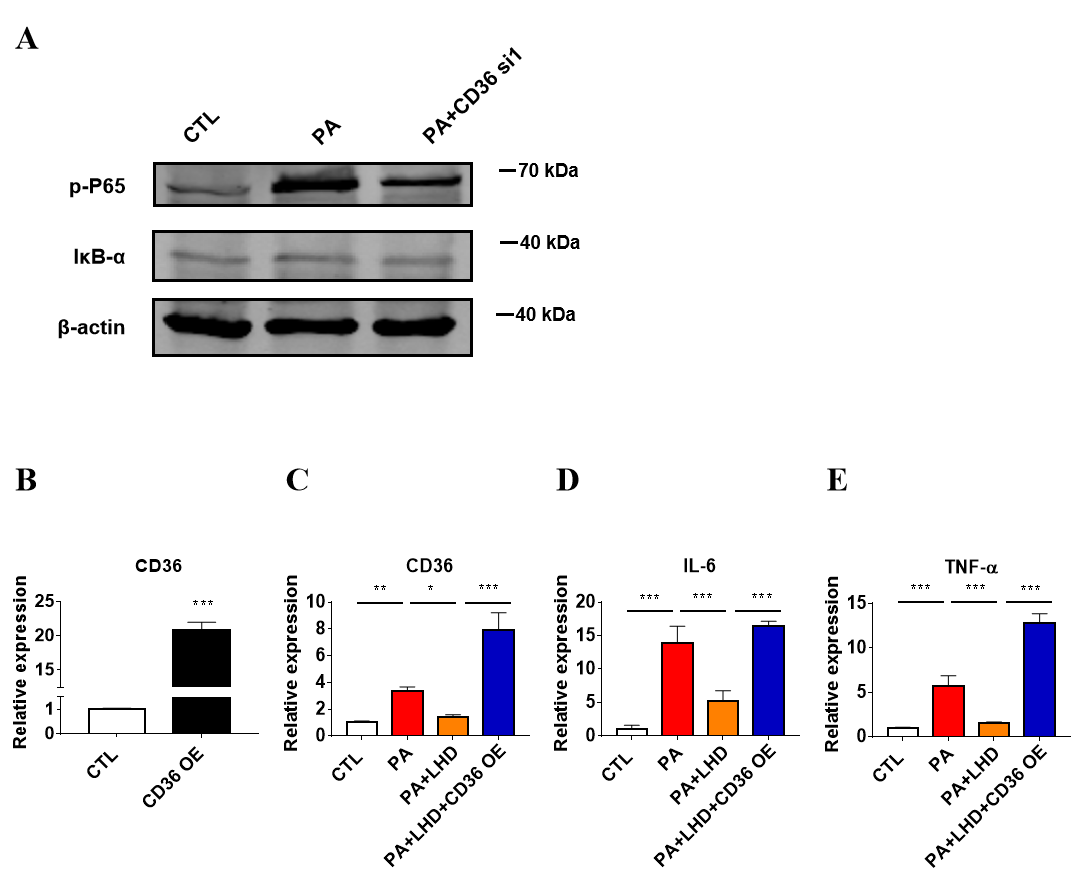


**Supplementary Figure 2：The role of CD36 in PA-induced cardiomyocyte inflammation.**

**(A)** Western blotting analysis of the expression of the p-P65 and IκB-α inflammatory signaling pathway proteins by CD36 siRNA treated human cardiomyocytes, *n* = 3. **(B)** Expression of C D36 mRNA after CD36 overexpression for 24 h in AC16 cells. ****P* <0.001, compared with the CTL group, *n* = 3. **(C)** Effect of CD36 overexpression on the expression of CD36 in PA-treated AC16 cells. **P* < 0.05, ***P* < 0.01, ****P* < 0.001, *n* = 3. **(D)** Effect of CD36 overexpression on the expression of IL-6 in PA-treated AC16 cells. ****P* < 0.001, *n* = 3. **(E)** Effect of CD36 overexpression on the expression of TNF-α in PA-treated AC16 cells. ****P* < 0.001, *n* = 3.


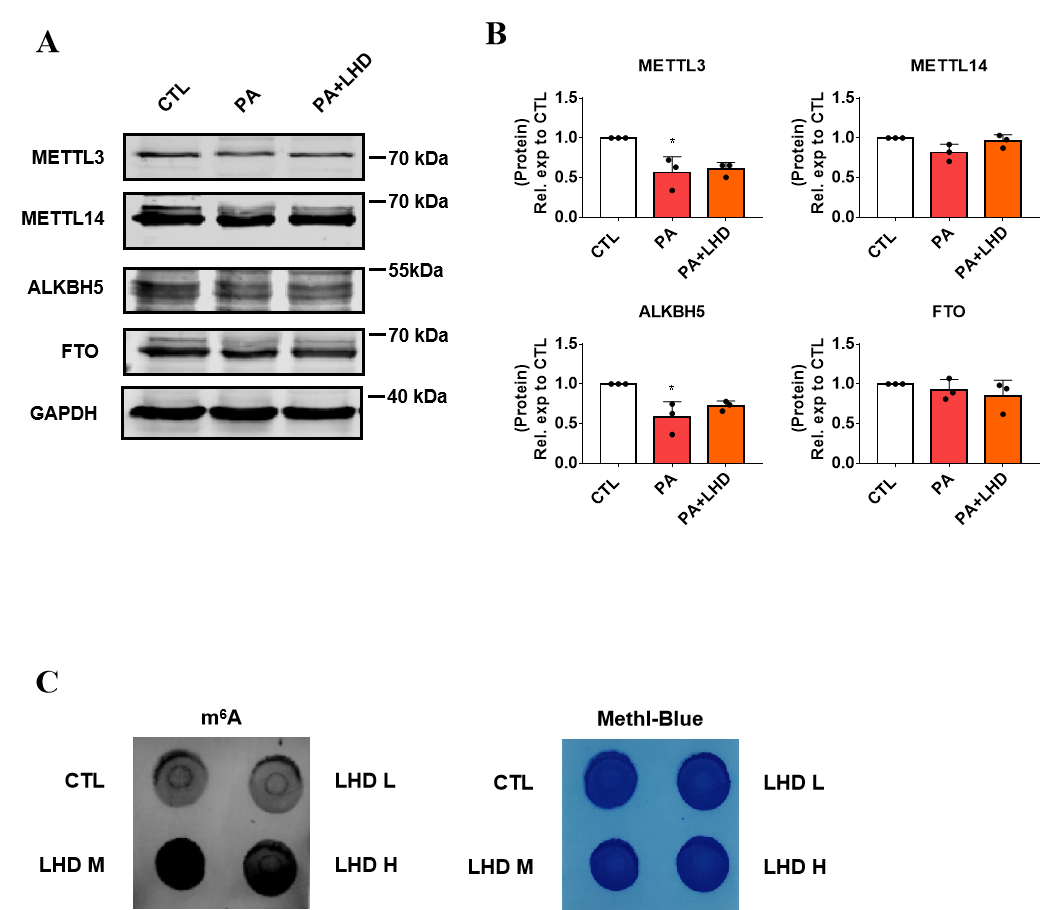


**Supplementary Figure 3：LHD regulates the level of m^6^A and methylation modifying enzymes.**

**(A-B)** Western blot detection of inflammatory signaling pathway FTO expression in human cardiac myocytes; (B) statistical plot of protein change results, **P* < 0.05, compared with the CTL group, *n =* 3. **(C)** Dot blot detection intracellular m^6^A levels after different concentrations of LHD-treated cells. CTL (0 µM LHD), LHD L (12.5 µM LHD), LHD M (25 µM LHD), LHD H (50 µM LHD), *n =* 3.

**
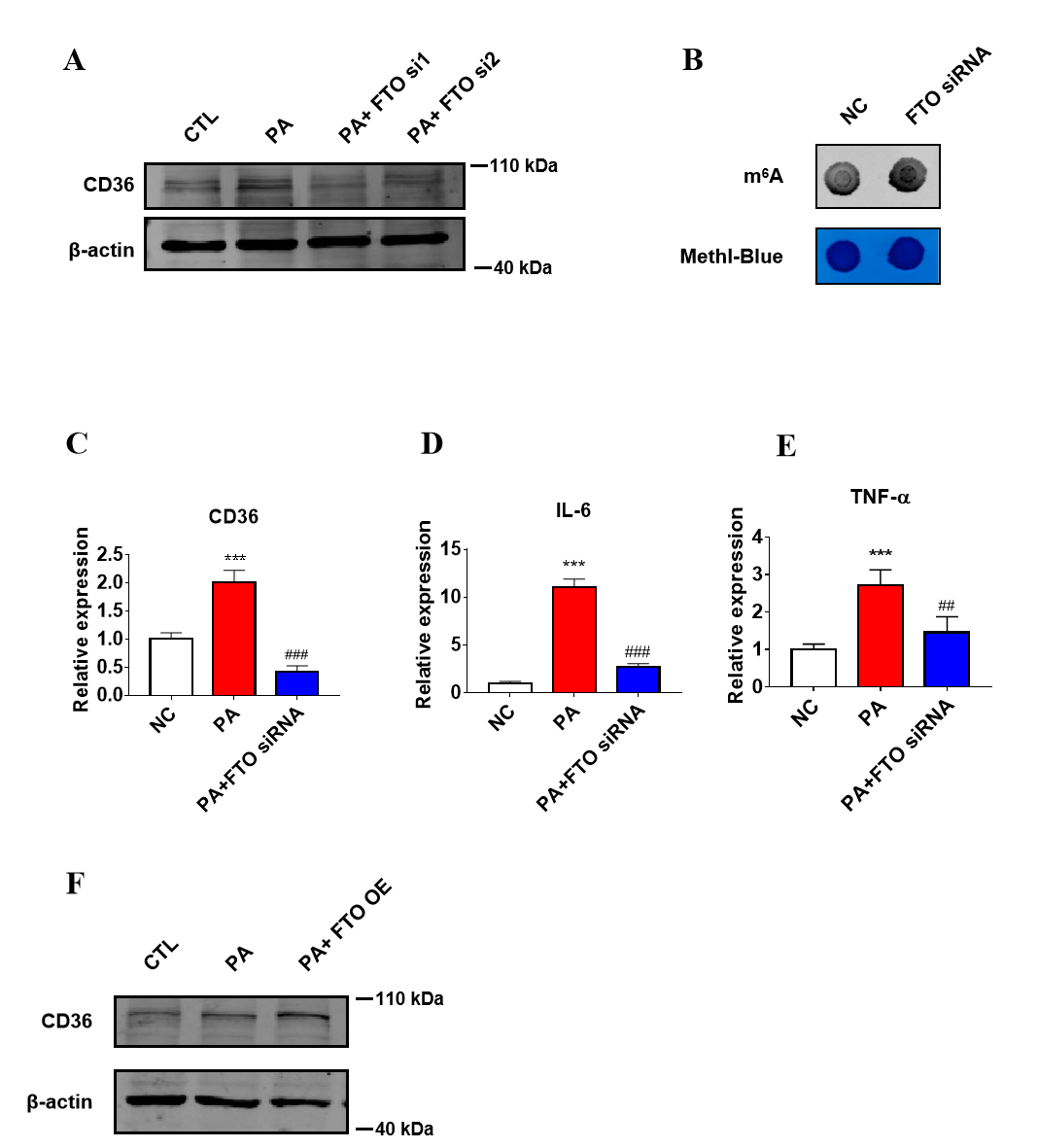
**

**Supplementary Figure 4 ：FTO silencing regulates m^6^A levels and inflammatory cytokines expression.**

**(A)** Western blot detection of CD36 expression by FTO si RNA treated human cardiac myocytes, *n =* 3.**(B)** Dot blot detection intracellular m^6^A levels in FTO siRNA transfected cells, *n* = 4. **(C)** Effect of FTO silencing on the expression of CD36 in AC16 cells. ****P* < 0.001, compared with the NC; ^###^*P* < 0.001, compared with the PA group, *n* = 4. **(D)** Effect of FTO silencing on IL-6 expression in AC16 cells. ****P* < 0.001, compared with the NC group; ^###^*P* < 0.001, compared with the PA group, *n* = 4. **(E)** Effect of FTO silencing on TNF-α expression in AC16 cells. ****P* < 0.001, compared with the NC group; ^##^*P* < 0.01, compared with the PA group, *n* = 4. **(F)** Western blot detection of CD36 expression by FTO overexpression plasmid treated human cardiac myocytes, *n =* 3.

**Supplement Table 1: The sequences of siRNAs.**

| Genes | Forward primer (5'-3') | Reverse primer (5'-3') |
| --- | --- | --- |
| FTO 1 | GUUCACAACCUCGGUUUAGTT | CUAAACCGAGGUUGUGAACCG |
| FTO 2 | ACCAAGGAGACUGCUAUUUTT | AAAUAGCAGUCUCCUUGGUGA |
| CD36 1 | AAACTTCTGAACATGTTTGCCCCTGTCTC | GAGACAGGGGCAAACAUGUUCAGAAGUUU |
| CD36 2 | AACACAGGGATTCCTTTCAGACCTGTCTC | GAGACAGGUCUGAAAGGAAUCCCUGUGUU |
| NC | UUCUCCGAACGUGUCACGUTT | ACGUGACACGUUCGGAGAATT |

**Supplement Table 2: Primer sequences used in Quantitative Real-Time PCR**

| Genes | Forward primer (5'-3') | Size（bp） | Reverse primer (5'-3') | Size（bp） | GenBank numbers |
| --- | --- | --- | --- | --- | --- |
| IL-6 | ATCCTCGACGGCATCTCAGC | 20 | GCCAGTGCCTCTTTGCTGCT | 20 | NM_000600.5 |
| TNF-α | CCCAGGGACCTCTCTCTAATC | 21 | ATGGGCTACAGGCTTGTCACT | 21 | NM_000594.4 |
| CD36 | CTTTGGCTTAATGAGACTGGGAC | 23 | GCAACAAACATCACCACACCA | 21 | NM_001001548.3 |
| FTO | AGCAGGTAATGTTCGGGCAAT | 21 | TGTGGAAGAAGATGGAGGGTG | 21 | NM_001080432.3 |
| ATP5MC | TTCCAGACCAGTGTTGTCTCC | 21 | GACGGGTTCCTGGCATAGC | 19 | NM_005175.3 |
| ATP5FID | ACTCTTCGGTGCAGTTGTTGG | 21 | GCCTCGATTCGGATCTGGAT | 20 | NM_001687.5 |
| HADHB | CTGTCCAGACCAAAACGAAGAA | 22 | CGATGCAACAAACCCGTAAGC | 21 | NM_000183.3 |
| ACADM | GGAAGCAGATACCCCAGGAAT | 21 | AGCTCCGTCACCAATTAAAACAT | 23 | NM_000016.6 |
| ACADVL | TCAGAGCATCGGTTTCAAAGG | 21 | AGGGCTCGGTTAGACAGAAAG | 21 | NM_000018.4 |
| SLC27a1 | GGGGCAGTGTCTCATCTATGG | 21 | CCGATGTACTGAACCACCGT | 20 | NM_198580.3 |
| CPT1b | CCTGCTACATGGCAACTGCTA | 21 | AGAGGTGCCCAATGATGGGA | 20 | NM_152246.3 |
| 18s | CCTGGATACCGCAGCTAGGA | 20 | GCGGCGCAATACGAATGCCC | 20 |  |

**Supplement Table 3: Top 15 proteins predicted bind to LHD.**

| Number | Target | Common name | ChEMBL ID | Target Class |
| --- | --- | --- | --- | --- |
| 1 | Protein kinase C gamma | PRKCG | CHEMBL2938 | Kinase |
| 2 | Protein kinase C beta | PRKCB | CHEMBL3045 | Kinase |
| 3 | Protein kinase C eta | PRKCH | CHEMBL3616 | Kinase |
| 4 | Alpha-ketoglutarate-dependent dioxygenase FTO | FTO | CHEMBL2331065 | Oxidoreductase |
| 5 | Protein kinase C alpha | PRKCA | CHEMBL299 | Kinase |
| 6 | Matrix metalloproteinase 13 | MMP13 | CHEMBL280 | Protease |
| 7 | Matrix metalloproteinase 9 | MMP9 | CHEMBL321 | Protease |
| 8 | Matrix metalloproteinase 1 | MMP1 | CHEMBL332 | Protease |
| 9 | Phosphodiesterase 5A | PDE5A | CHEMBL1827 | Phosphodiesterase |
| 10 | Tubulin beta-1 chain | TUBB1 | CHEMBL1915 | Structural protein |
| 11 | Hepatic lipase | LIPC | CHEMBL2127 | Enzyme |
| 12 | Matrix metalloproteinase 3 | MMP3 | CHEMBL283 | Protease |
| 13 | Cyclin-dependent kinase 2 | CDK2 | CHEMBL301 | Kinase |
| 14 | Cyclin-dependent kinase 4 | CDK4 | CHEMBL331 | Kinase |
| 15 | ADAM17 | ADAM17 | CHEMBL3706 | Protease |
